# Transcatheter Closure of Complex Multifenestrated Atrial Septal Defect Using Three Septal Occluders: Is Three Too Many?

**DOI:** 10.1016/j.shj.2024.100355

**Published:** 2024-09-05

**Authors:** Hussam Al Hennawi, Appa Bandi, Ahmad Abulshamat, Shaurya Srivastava, Mohammed Qintar

**Affiliations:** a Department of Internal Medicine, Jefferson Abington Hospital, Abington, Pennsylvania; b Department of Cardiology, University of Michigan Health – Sparrow Heart and Vascular, and Michigan State University, Lansing, Michigan; c Division of Cardiology, Jordan University Hospital, Amman, Jordan

**Keywords:** Amplatzar septal occluder, Atrial septal defect, Fenestrated atrial septal defects

## Abstract

Atrial septal defects (ASDs) are a common form of adult congenital heart disease, with surgical or transcatheter closure being the mainstay of treatment in patients with hemodynamic or clinical complications. Transcatheter repair of secundum ASD is safe and effective. Complex secundum ASDs with multifenestrated defects pose a unique challenge to transcatheter repair. We report a successful but complex transcatheter closure of an ASD using three overlapping Amplazter devices.

Atrial septal defects (ASD) are a common adult congenital heart disease, typically treated by surgical or transcatheter closure, with transcatheter repair being safe and effective for secundum ASDs, though complex, multi-fenestrated defects pose unique challenges.^1^, ^2^, ^3^ A 55-year-old male with a history of familial hypercholesterolemia, paroxysmal atrial fibrillation, and chronically occluded right coronary artery presented for atrial fibrillation ablation and was found to have a large and complex multifenestrated ASD with dilated right ventricle and a large shunt. The patient was asymptomatic except for mild shortness of breath on presentation.

Echocardiogram demonstrated aneurysmal intra-atrial septum with evidence of left to right shunt and moderate dilation of the right atrium and right ventricle. Cardiac magnetic resonance showed severe biatrial enlargement with aneurysmal multifenestrated interatrial septum with Qp/Qs of >2. A transesophageal echocardiogram also demonstrated an aneurysmal and multifenestrated complex interatrial septum with a discernable secundum ASD noted with three distinct defects and a possible patent foramen ovale (Videos 1, 2, and 3). Flow Doppler demonstrated mostly left to right shunt (Video 1). The patient elected for a transcatheter approach utilizing Amplatzer Septal Occluder (Saint Jude Medical, St Paul, MN).

A right heart catheterization was done and demonstrated right atrial pressure of 11 mmHg, right ventricular: 41/1 mmHg, pulmonary artery: 40/17/28 mmHg, pulmonary capillary wedge pressure: 14 mmHg, pulmonary vascular resistance: 142 dynes/sec/cm, a mixed venous saturation of 74%, inferior vena cava: 77%, RV/PA: 89%, and aorta: 98% with Qp/Qs of 2.5. Under fluoroscopic and transesophageal echocardiogram (TEE) guidance, we deployed a 35-mm Amplatzer cribriform septal occluder in the patent foramen ovale; a 12-mm and a 24-mm Amplatzer Septal Occluder were deployed in 2 of the 3 ASDs with no residual flow to the right atrium by color Doppler on TEE (Videos 4 and 5). The third and very small inferior ASD was left without closure due to a deficient posterior rim covered by the disk of the other devices, and there was negligible flow across it. Postprocedure right heart catheterization demonstrated an right ventricular oxygen saturation of 67% (89% before intervention) with a Qp/Qs of 1. The three Amplatzer devices were then successfully released. Final TEE color flow Doppler images demonstrated a very small residual left-to-right atrial shunt (Video 6).

## Consent Statement

Consent was obtained from the patient to publish this report and any accompanying images.

## Funding

The authors received no funding to disclose.

## Disclosure Statement

The authors have nothing to disclose.

## Supplementary Material

Video 1 Transesophageal echocardiogram demonstrating an aneurysmal and multifenestrated complex interatrial septum.

Video 2 3D transesophageal echocardiogram demonstrating multifenestrated complex interatrial septum.

Video 3 Transesophageal echocardiogram with X-plane through the septum showing the PFO along with the atrial septal defect.

Video 4 Fluoroscopy showing the overlapping three Amplatzer Occluders.

Video 5 3D transesophageal echocardiogram showing the three Amplatzer Occluders from both the right and left atrial point of view.

Video 6 Transesophageal echocardiogram with color showing no residual shunt through the septum post closure.
